# Visualizing the Indefinable: Three-Dimensional Complexity of ‘Infectious Diseases’

**DOI:** 10.1371/journal.pone.0123674

**Published:** 2015-04-14

**Authors:** Gabriel Leitner, Shlomo E. Blum, Ariel L. Rivas

**Affiliations:** 1 National Mastitis Reference Center, Kimron Veterinary Institute, Bet Dagan, Israel; 2 Center for Global Health, Internal Medicine, Health Sciences Center, University of New Mexico, Albuquerque, New Mexico, United States of America; 3 Population Health and Pathobiology, North Carolina Sate University, Raleigh, North Carolina, United States of America; Cardiff University School of Medicine, UNITED KINGDOM

## Abstract

**Background:**

The words ‘infection’ and ‘inflammation’ lack specific definitions. Here, such words are not defined. Instead, the ability to visualize host-microbial interactions was explored.

**Methods:**

Leukocyte differential counts and four bacterial species (*Staphylococcus aureus*, *Streptococcus dysgalactiae*, *Staphylococcus chromogenes*, and *Escherichia coli*) were determined or isolated in a cross-sectional and randomized study conducted with 611 bovine milk samples. Two paradigms were evaluated: (i) the classic one, which measures non-structured (count or percent) data; and (ii) a method that, using complex data structures, detects and differentiates three-dimensional (3D) interactions among lymphocytes (L), macrophages (M), and neutrophils (N).

**Results:**

Classic analyses failed to differentiate bacterial-positive (B+) from –negative (B−) observations: B− and B+ data overlapped, even when statistical significance was achieved. In contrast, the alternative approach showed distinct patterns, such as perpendicular data inflections, which discriminated microbial-negative/mononuclear cell-predominating (MCP) from microbial-positive/phagocyte-predominating (PP) subsets. Two PP subcategories were distinguished, as well as PP/culture-negative (false-negative) and MCP/culture-positive (false-positive) observations. In 3D space, MCP and PP subsets were perpendicular to one another, displaying ≥91% specificity or sensitivity. Findings supported five inferences: (i) disease is not always ruled out by negative bacterial tests; (ii) low total cell counts can coexist with high phagocyte percents; (iii) neither positive bacterial isolation nor high cell counts always coincide with PP profiles; (iv) statistical significance is not synonymous with discrimination; and (v) hidden relationships cannot be detected when simple (non-structured) data formats are used and statistical analyses are performed before data subsets are identified, but can be uncovered when complexity is investigated.

**Conclusions:**

Pattern recognition-based assessments can detect host-microbial interactions usually unobserved. Such cutoff-free, confidence interval-free, gold standard-free approaches provide interpretable information on complex entities, such as ‘infection’ and ‘inflammation’, even without definitions. To investigate disease dynamics, combinations of observational and experimental longitudinal studies, on human and non-human infections, are recommended.

## Introduction

The word ‘infection’ has been used for centuries. When the terms ‘infection’ and ‘definition’ are searched for, the *Web of Science* currently retrieves more than 7000 publications. Hence, readers might expect to find a large consensus on the definition of ‘infection.’ Yet, nothing could be farther from the truth: more than 700 definitions have been proposed for just one disease [1]. Such situation is seen in many diseases [2]. If no agreement can be reached on the definition of a single infectious disease, how can infections be diagnosed?

After 132 years, Koch’s postulates still apply: infectious diseases are caused by microbes, which may be isolated [3]. However, that is only one of many possibilities. Even Koch modified his first postulate: infection differs from colonization [3]. Similarly, a bacterial-negative test is no evidence of an infection-free status: false-negative tests (infections in which no bacterium is detected) may occur due to a variety of reasons [4, 5]. Because infections have at least six expressions [6] and because, historically, diagnosis has been based on a circular fallacy (to select a diagnostic test, the identity of the infecting microbe should be known in advance and, to identify a specific microbe, a specific diagnostic test is needed), it seems that diagnosing ‘infection’ cannot be achieved with classic approaches—a different paradigm is needed.

To visualize infectious diseases, its ‘mirror image’—the immune response—may be considered. While not specific, immunity is not prone to the problems described above [7, 8]. However, both ‘immunity’ and ‘inflammation’ lack a single, agreed-upon definition. Instead of focusing on definitions, here we focus on the data used to diagnose and prognosticate.

While the *technology* used to collect data is not a major problem, data *interpretation* is problematic. Interpretation involves several issues, such as: (i) *data structuring*; (ii) unintended consequences of *combinatorial interactions* (e.g., *errors*); and (iii) a topic encapsulated in this question: are host-microbial interactions *complex* or *simple* systems?

In clinical medicine, information refers to separate what is different, i.e., to discriminate. Discrimination is the requisite for medical decisions. To decide, data should be transformed into interpretable information [9]. Such process requires *data structuring*.

To illustrate the difference between data and information, let us consider a ‘*bio-economical’* question: how to sustain life at the lowest cost—that is, how to do more, with less, better and faster? Because the number of pathogens is too large to be known, but the number of immune cell types is very low (up to 5 cell types, according to classic immunology; or less than 300 cell types [10], if we assume that all cell types may participate in immune responses), it follows that a one-to-one relationship (one specialized cell type per each pathogen) is not adequate—if such strategy had been adopted, all creatures would be much larger than the largest elephant, becoming highly vulnerable to any food shortage. Instead, evolution has conserved small creatures. How can small creatures face so many pathogens with so limited resources?

The answer involves *biological combinations* [11, 12]. The same element—combined with other element(s)—can perform many (including opposite) functions. For instance, monocytes both promote and destroy neutrophils. Similarly, some cytokines induce and prevent the same immune response [13–16].

While combinations foster survival, they also create problems, such as *non-interpretable* data and *hidden interactions* [17]. Unless shown otherwise, biological data may resemble an iceberg: we only see its tip. While valuable information may be hidden, the observed data may be *non-interpretable* and/or *error*-prone. For example, when a numerical cut-off is imposed on continuous data (such as cell counts) and discontinuous (discrete) labels are assigned to data points located above or below the cut-off (e.g., infected and non-infected), it has been known, since 1983, that false-negatives and/or false-positives will be generated [18].

Because all elements of the immune system are always interacting and changing, the unit of study is not any one observable data point, but the usually unobserved system [19]. Then, is the immune system *simple* or *complex*? Is it internally homogeneous or heterogeneous? Is it composed of only one level or its functions involve multiple levels? The literature indicates that it is complex, heterogeneous, and multi-level [12, 20, 21].

What does ‘complex’ actually mean? First, it means that there are numerous and heterogeneous elements (e.g., cell receptors), which perform different *functions* [22]. Second, it means that it is composed of many structural *levels* (e.g., organs). The mathematics that suit one level may differ from that of another level [12]. Such levels also differ in their *contents*. For instance, human blood only contains approximately 2.2% of all lymphocytes, the bone marrow ~ 10%, the gut between 5 and 20%, and, together, the spleen and lymph nodes contain about 60% lymphocytes [23]. In addition, different *relationships* may occur: while the CD4/CD8 lymphocyte ratio is about 1.2:1 in lymph nodes, it is 0.3:1 in the spleen [23]. Third, it means that a complex system is *interdependent*, with communication flows going in all directions [22].

Because the immune system is not a static, internally homogeneous, single-level, single-flow oriented structure composed of independent elements which always have the same meaning (and it may include unobserved interactions), inferences or predictions about the immune system cannot be based on ‘sampling’ any one element. The basic tenets of classic statistical tests (data independence and normality) do not apply to host-microbial interactions [24]. If ‘random sampling’ does not apply to infectious diseases, how can they be assessed?

Uncovering complexity is one alternative. Methods that reveal biological complexity may extract more information than classical approaches [25]. Complex systems are characterized by three properties: (i) emergence, (ii) irreducibility, and (iii) unpredictability [26–28].

*Emergence* (also known as *novelty*) refers to the *new* features revealed only when a complex (system-level) structure is assembled. *Irreducibility* means that *emergence* cannot be explained by or reduced to the properties of any one variable. *Unpredictability* refers to the inability to predict emergence when only ‘simple’ and/or isolated variables are analyzed, e.g., immunoglobulins express emergent properties, which are neither reducible to first principles nor predictable [29]. Similarly, the three-dimensional (3D) functions performed by cell groups (multi-cellularity) cannot be predicted by 2D models—much less by 1D or tabular formats [30].

To elicit the hidden information complex systems tend to have, dimensionless indicators can be considered [31, 32]. Such indicators, together with data structures designed to possess some desirable properties, could provide pattern recognition-based information [33, 34].

Here we explored a proof-of-concept derived from the previous considerations, which was evaluated with microbiological and leukocyte data collected from animals. Two questions were asked: (i) do host-microbial interactions reveal complex properties?, and (ii) if demonstrated, can such properties provide information that facilitate medical decisions?

## Materials and Methods

### Animals

A cross-sectional and randomized study was performed with 611 bovine milk samples collected from the same number of mammary glands in mid-lactating Israeli Holstein cows. Samples were used to identify and quantify leukocytes, and conduct microbial cultures (S1 Dataset). No bacteria were found in 464 of such samples, and three bacterial species were isolated from the remaining 147 quarters: (i) *Staphylococcus aureus* (n = 31); (ii) *Staphylococcus chromogenes*, (n = 74); and (iii) *Streptococcus dysgalactiae* (n = 42). Seventy eight culture-negative tests proceeded from mammary glands that, one month earlier, yielded *Escherichia coli* isolates. *E*. *coli*+ cows were treated, for 3 days, with antibiotics (ABIC Biological Laboratories, Teva Ltd., Israel) and anti-inflammatory drugs (Norbrook Laboratories, Ireland). No bacterium was isolated in the previous month from milk of the remaining cows. Data analyses were conducted twice: not considering and (only in the case of animals previously *E*. *coli*-positive, which were treated) considering prior information. While cross-sectional in a strict sense, the design used provided more information than classic cross-sectional designs because it also considered prior data.

This study was approved by the Institutional Animal Care Committee of the Israeli Agricultural Research Organization of the Volcani Center and the Institutional Animal Care and Use Committee of the University of New Mexico, United States (protocol 13-101022-T-HSC).

### Bacteriological and leukocytes count methods

Culture and molecular procedures with a detection limit estimated a 10 colony-forming units/ml were performed as described before [35]. Negative samples were re-tested with a kit that extracts bacterial DNA using universal 16S primers 4F and 801R (Milk Bacterial DNA Isolation Kit, Norgen Biotek Corp., Ontario, Canada [36]). Total milk cell counts/ml (‘somatic cells’ counts or SCC) were determined with a Fossomatic 360 counter (Foss Electric, Hillerod, Denmark). Usng the technique and monoclonal antibodies anti-bovine CD18, CD4, CD8, CD14 and G1 described earlier [37], each leukocyte was identified and its percentage calculated using a FACSCalibur cytometer (Becton-Dickinson Immunocytometry System, San Jose, CA, USA). Because SCC include epithelial cells, leukocyte counts were determined as CD18^+^ counts/ml.

### Leukocyte data structuring

Primary leukocyte data on lymphocytes (L), macrophages/monocytes (M), and neutrophils (N) were used to *detect data subsets* that, in 3D space, displayed *distinct patterns* (e.g., perpendicular data inflections). To that end, a three-step method was implemented: (i) *dimensionless indicators were created*, which expanded the number of data structures available for analysis; (ii) 3D data structures lacking distinct patterns *were deleted*; and (iii) using leukocyte and bacteriological data, the *biological validity of each distinct data subset was explored*. This process was facilitated by a proprietary algorithm that creates dimensionless indicators (combinations of two or more cell types as ratios or products derived from counts or percentages, alone or combined). Biological validity was supported when two or more data combinations identified two or more subsets, which displayed non-overlapping data distributions and differed in discrete classifiers (e.g., when culture-negative observations predominated in one data subset and culture-positive data points predominated in the other subset).

### Data analyses

Dimensionless indicators (DIs) were generated as described elsewhere [31, 32]. DIs were temporary guides used to detect distinct data subsets. Because DIs can include many, internally complex relationships (e. g., the ([M% * N%] * [M counts * N counts]) / ([MC/N] / [N%+M%]/ L%]), and the patterns they generate are not the result of any one indicator but the overall 3D interaction, such patterns cannot be predicted. Consequently, the internal structure of individual DIs—identified here with letters expressed in italics, e.g., *BAL*—was not described. Instead, the data contents of subsets detected by DIs were described. When one or more data inflections distinguished two or more data subsets, the L, M, and N counts or percentages of each subset were explicitly analyzed. Such evaluation also explored indices that reflect either early anti-microbial responses (e.g., the neutrophil/lymphocyte [N/L] ratio, which increases in early responses) or later responses (e.g., the mononuclear cell/N [MC/N] ratio, which increases in the resolution phase) [38, 39]. Statistical analyses of medians (Mann-Whitney test) or proportions (Chis square test), as well as 3D plots, were conducted or created with a commercial package (Minitab Inc, State College, PA, USA).

## Results

**Fig 1:**
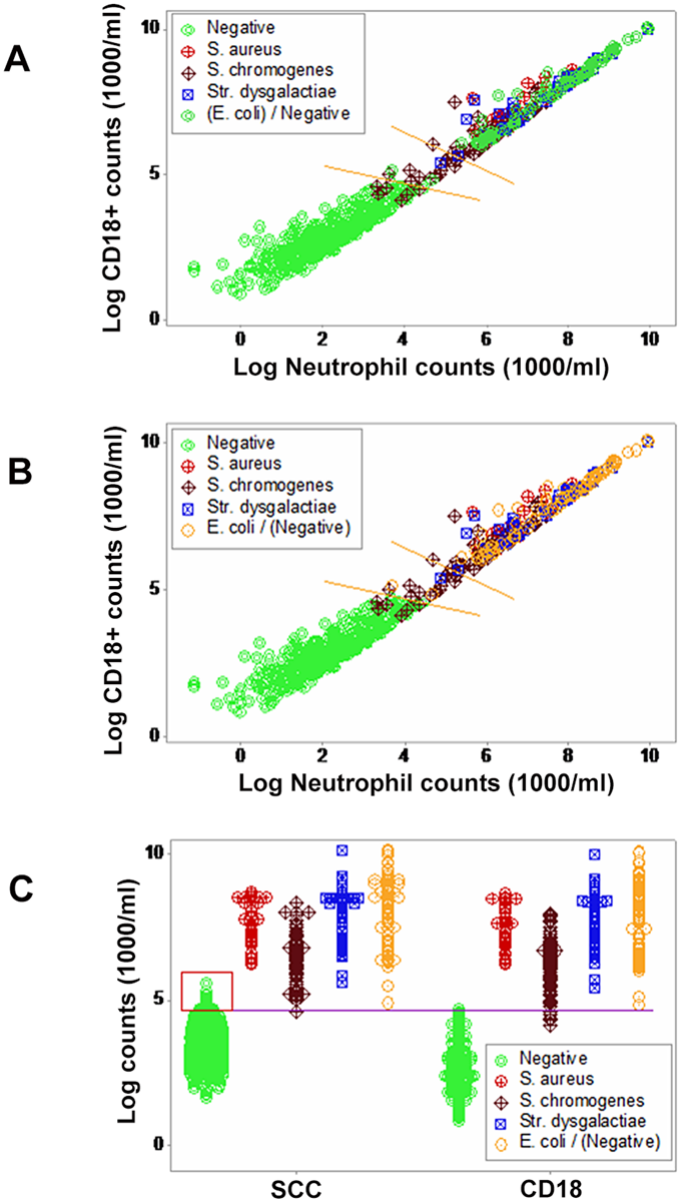
Discriminant diagnostic ability of cell counts. Cell counts collected from 611bovine milk samples are reported. When CD18+ and neutrophil (N) counts were assessed and cultures were conducted, culture-negative observations predominated below (log) 5 CD18+ counts/ml; however, numerous culture-negative data points were also observed above such threshold (**A**). Culture-negative observations above (log) 5 (CD18+ 1000 counts/ml) corresponded to animals previously *E*. *coli*-positive. If prior information were considered, most observations above (log) 5 CD18 would become positive (**B**). Because animals previously *E*. *coli*-positive were treated with antibiotics and anti-inflammatory drugs (one month before the leukocyte profile was conducted) and in both instances the same bacteriological procedures were implemented, the discrepancy observed (high total cell counts, predominantly explained by high neutrophil counts, in 61 culture-negative samples of the later assessment) cannot be attributed to either the bacteriological procedures (the same, in both instances) or to the absence of antibiotic and anti-inflammatory treatment. When prior information was considered, CD18+ counts discriminated better that the SCC: CD18+ counts did not overlap between culture-negative and—positive observations but revealed a substantial overlapping when SCC were utilized (red box, **C**). Therefore, the use of counts, if considered alone (without considering prior information on culture results and treatments), is not diagnostic: counts would display the pattern shown in **A**.

The total leukocyte count revealed a threshold around (log) CD18+ count = 5. While culture-negative results predominated below that value, 78 culture-negative observations were found above such threshold (Fig 1A). Prior information seemed relevant: had prior information been considered (animals previously *E*. *coli-*positive, which were culture-negative when leukocytes were measured), negative observations observed at (log) CD18+ counts >5 would become positive (Fig 1B). Prior information demonstrated that CD18+ counts were better indicators than the SCC: while a non-trivial number of culture-negative and—positive observations overlapped when SCC were measured (red box, Fig 1C), CD18 counts showed negligible data overlapping (Fig 1C).

**Fig 2:**
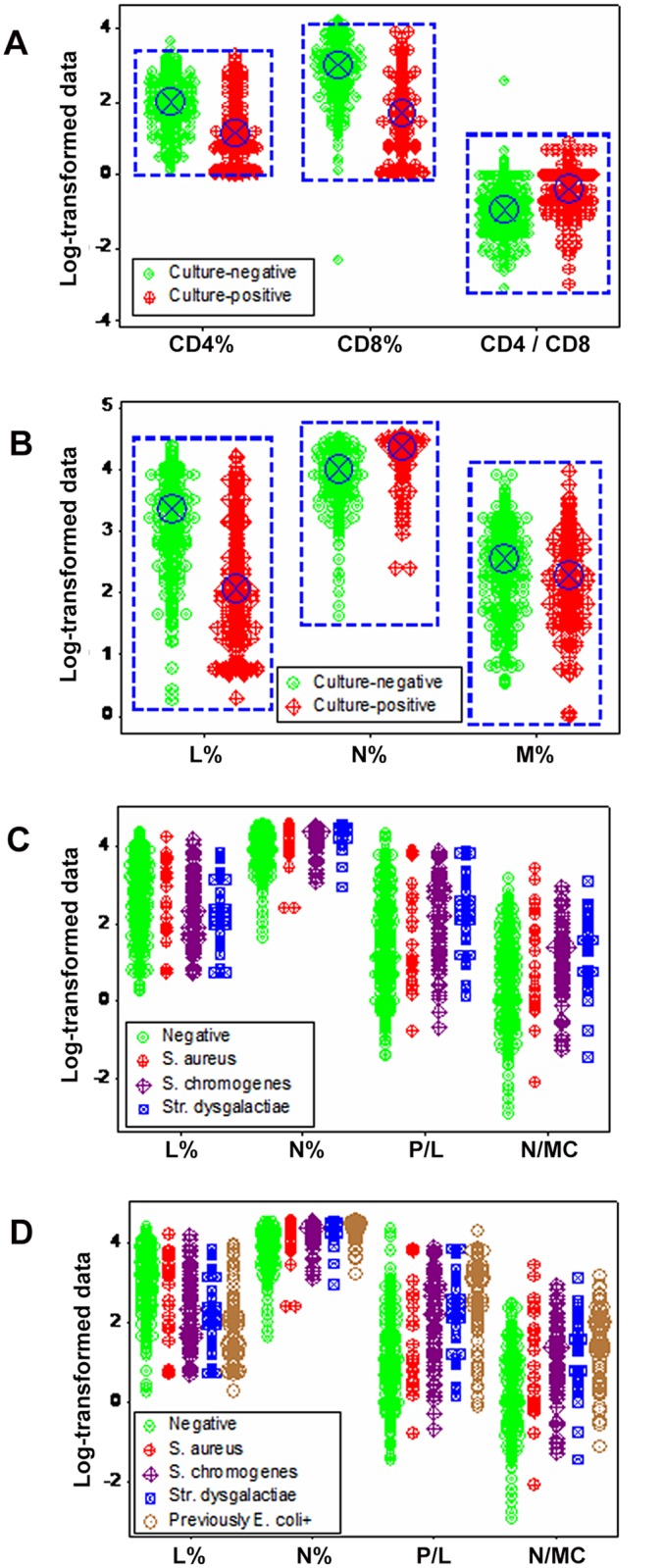
Discriminant diagnostic ability of simple or low-complexity indicators. Neither molecular (cell-surface marker-related) nor cellular indicators (expressed as leukocyte percentages) distinguished culture-negative from-positive observations (**A, B**). While median values reached statistical significance (blue circles, *P*<0.03, Mann-Whitney test, **A, B**), overlapping leukocyte data distributions were observed between the culture-negative and -positive groups (blue boxes, **A, B**). Such lack of discrimination remained when low-complexity indicators (ratios that measured interactions involving two or more cell types) were considered, regardless of current information on bacterial status (**C**) or prior information (i.e., assuming as culture-positive all previously *E*. *coli*-positive animals, **D**).

The fact that prior information is not always available prompted additional questions, such as: (i) is it possible to distinguish immune subcategories, if they exist?, (ii) can false culture-negatives and false culture-positives be detected?, (iii) can the same microbe induce different immune responses?, and (iv) is statistical significance synonymous with discrimination? None of such questions were answered by molecular data: the CD4+ lymphocyte percent, the CD8+ L%, and the CD4/CD8 ratio failed to distinguish culture-negatives from-positives, even when statistical significance was achieved (*P*<0.03, Fig 2A). Similarly, cellular data (L, N, or M percents) did not discriminate, even when median values differed statistically between the culture-negative and -positive groups (blue circles, *P*<0.03, Fig 2B). Regardless of prior information, neither cellular percentages nor indices that captured interactions involving two or more cell types (supra-cellular relationships) differentiated bacterium-specific infections: overlapping data distributions were observed (Fig 2C and 2D). Thus, regardless of the scale investigated, neither leukocyte- nor bacterial-related classes were distinguished when immunity was assumed to be a simple system, i.e., when L, M, or N data points were assessed separately.

**Fig 3:**
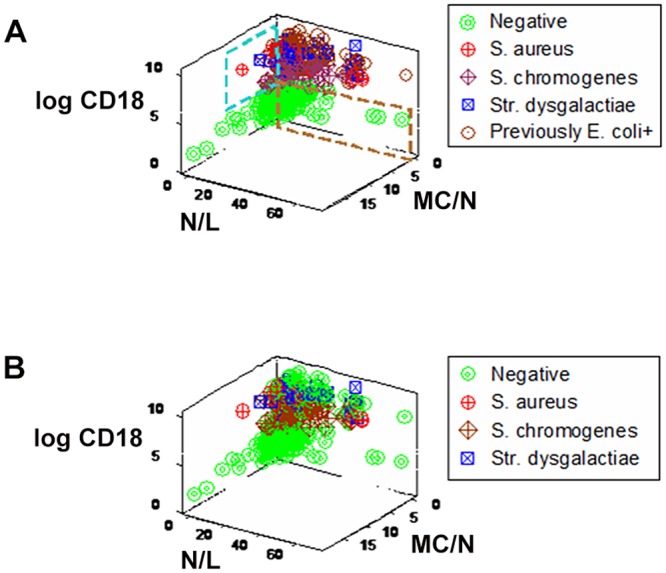
Discriminant diagnostic ability of simple or low-complexity indicators measured in 3D space. Cell count-based analyses, even when 3D patterns were considered, did not discriminate: CD18+ counts, together with the N/L and MC/N ratios, failed to distinguish immuno-microbial subsets when prior (**A**) or current (**B**) information was considered.

The use of counts, even when analyzed in 3D space, did not detect hidden interactions. For instance, when CD18+ counts were measured together with the N/L and MC/N ratios (indicators that characterize the early and late anti-microbial immune phases, respectively), both high N/L and high MC/N observations were associated with culture-negative and -positive observations (brown and blue boxes, respectively, Fig 3A). Discrimination was even poorer when current information was considered (Fig 3B).

**Fig 4:**
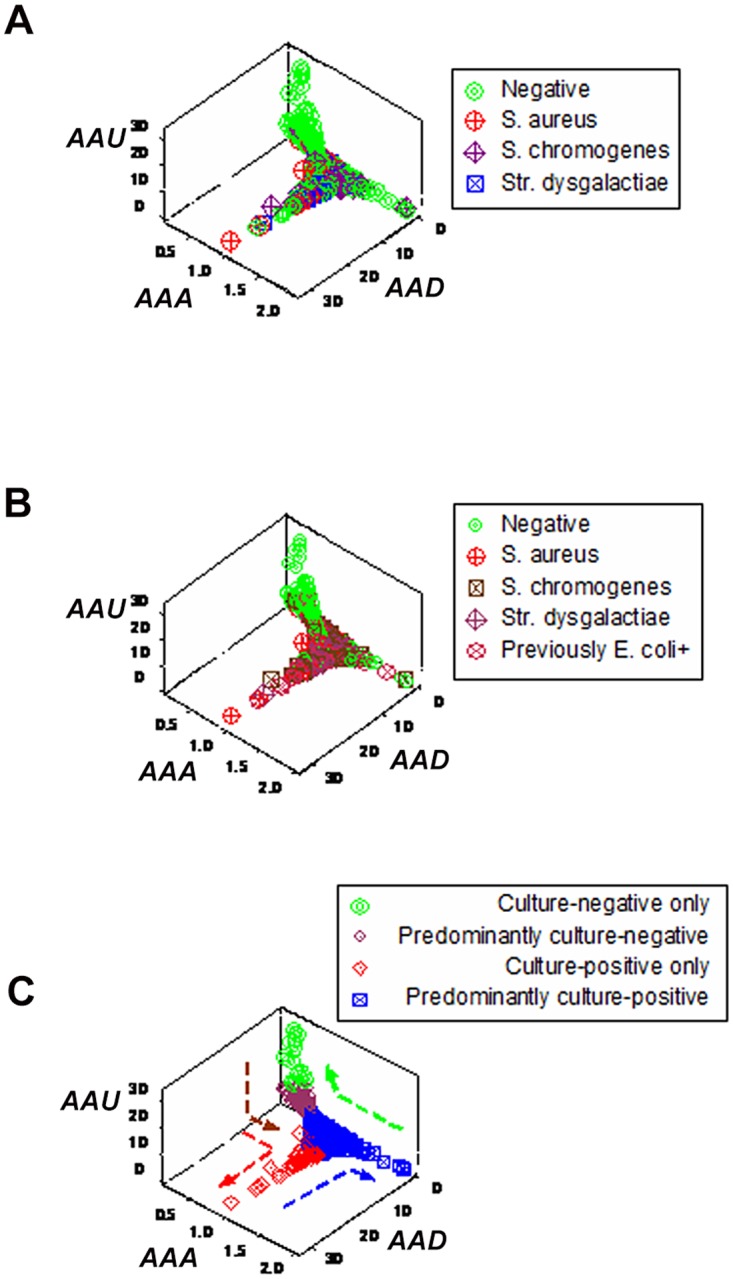
Discriminant diagnostic ability of complex, 3D data structures (emergence I). When dimensionless indicators were built and explored in three-dimensional (3D) space, distinct (perpendicular) data inflections (not previously observed) distinguished several subsets, demonstrating emergence (**A**). Emergent patterns were also found when prior information was considered (animals previously *E*. *coli*—positive were regarded to be positive, **B**). When prior information was considered, four perpendicular data inflections differentiated four subsets, characterized by: (i) culture-negative only, (ii) predominantly culture-negative, (iii) culture-positive only, and (iv) predominantly culture-positive observations (**C**).

In contrast, when dimensionless indicators were used and prior information was considered, *emergence* was demonstrated: four new data subsets were detected (Fig 4A and 4B). Data inflections characterized such subsets as: (i) culture-negative, (ii) predominantly culture-negative (including a few culture-positive data points), (iii) culture-positive, and (iv) predominantly culture-positive (including a few culture-negative observations, arrows, Fig 4C).

**Fig 5:**
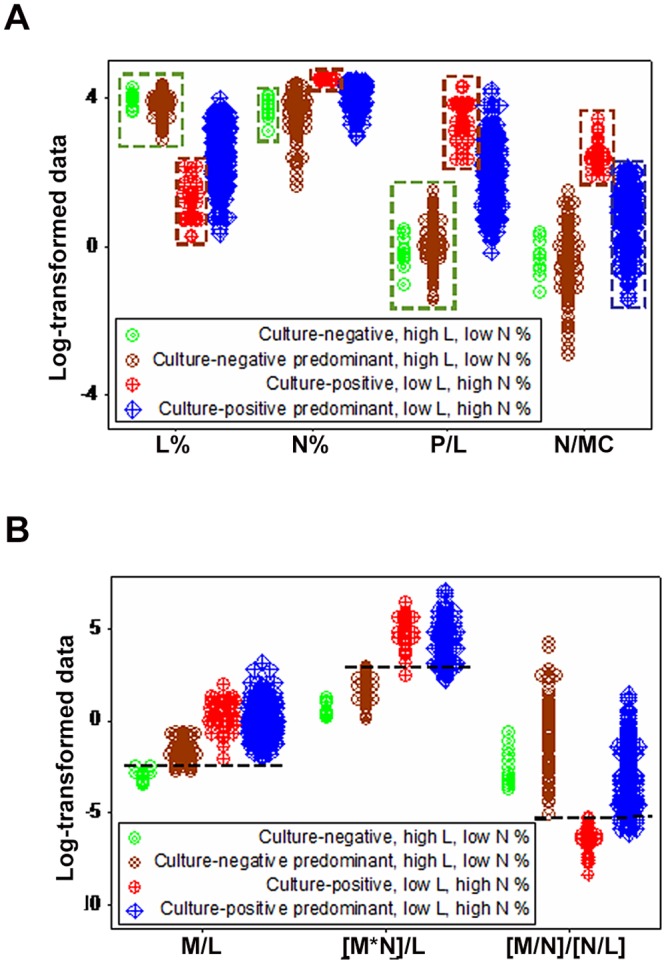
Data subset-validation, based on microbial-immunological data. Data subsets identified on the bases of dimensionless indicators (hypothetical indices of unknown biological validity) were evaluated with biologically explicit data. When prior information was considered, the culture-negative subsets showed the highest L %, while the culture-positive subset exhibited the lowest L%, and the highest N% (**A**). A similar pattern was observed when relationships involving monocytes and lymphocytes were analyzed; for instance, (i) the monocyte/lymphocyte (M/L) ratio distinguished the culture-negative, high L% subset from all other subsets; (ii) a composite index that included a product and a ratio (the [M*N]/ L) differentiated both culture-negative from both culture-positive subsets, and (iii) a double interaction (the [M/N] / [N]L] ratio) separated the low L%/high N%/culture-positive from all other subsets (lines, **B**).

When only current information was considered (i.e., when previously *E*. *coli*-positive cows were regarded as culture-negative—their status at testing time), data combinations improved discrimination. Metrics that, before, did not differentiate (Fig 2C and 2D), when re-grouped distinguished (with minor data overlapping) between culture-negative/mononuclear cell-predominating (MCP) subsets and culture-positive/phagocyte-predominating subsets (PP, boxes, Fig 5A). Additional indices (which included ratios and products) discriminated two phagocyte-related subsets: one totally and one only partially associated with positive cultures (red and blue symbols, Fig 5B).

**Fig 6:**
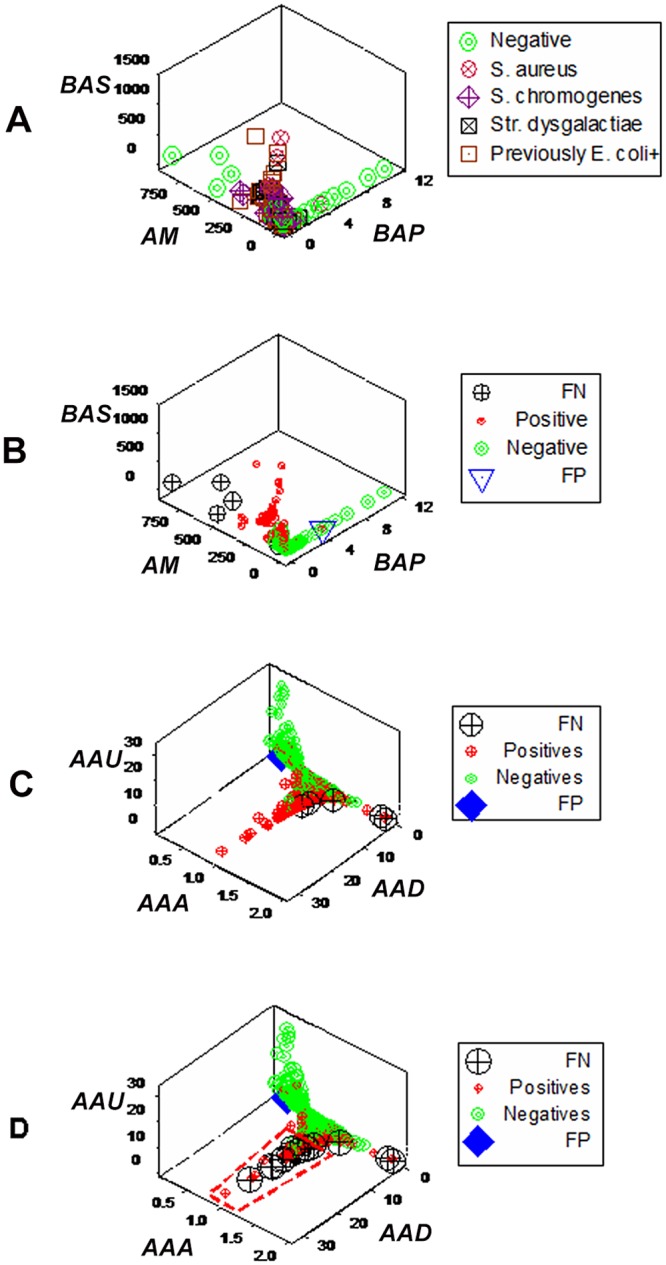
Detection and evaluation of observations suspected to be false (emergence II) Emergence was also expressed as observations suspected to be false. Such detection was elicited by dimensionless indicators (hypothetical indices derived from products or ratios of leukocyte data, which assess numerous relationships, e.g., *BAS*, *AM*, *BAP*). Dimensionless indicators detected two subsets of culture-negative data (**A**). Because culture-negative data subsets were separated by the culture-positive cluster, one subset was suspected to be false-negative (FN culture results, **B**). A similar contrast led to suspect that one culture-positive observation was a false-positive (FP): it was located within the culture-negative cluster but far from the location of culture-positives (**B**). Such inferences did not depend on the set of dimensionless indicators analyzed or whether current or prior information was considered: when a separate set of indicators was analyzed, it showed similar patterns (**C**) and, when only current information was considered, patterns remained, differing only in the number of FNs observed, which increased (red polygon, **D**).

Additional emerging patterns detected observations suspected to be false. When bacterial and leukocyte data were considered, one false-positive (FP or culture-positive/MCP) observation and five false-negatives (culture-negative/PP observations) were found (Fig 6A and 6B). False-negatives were located within the range of culture-positive observations, but far from the location of culture-negative/MCP data points (Fig 6C). The FP observation was located within the data range of culture-positives, but far from the location of culture-positives (Fig 6C). When prior information was not considered, additional FNs were detected (box, Fig 6D).

**Fig 7:**
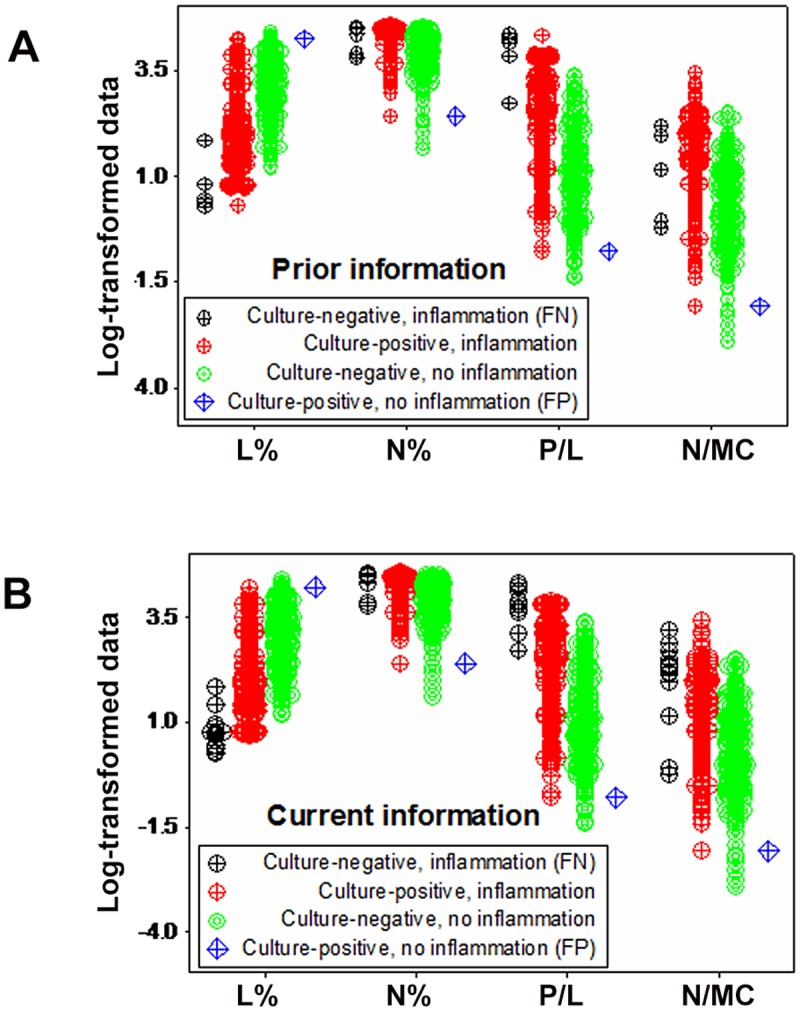
Pattern recognition-based differentiation. When hypothetical patterns were evaluated, four biologically different data subsets emerged when prior information was considered (A), which were characterized as: (i) culture-negative/phagocyte predominant (a subset displaying the lowest L% and high N%, i.e., the FN subset); (ii) culture-positive/mononuclear cell-predominant (a subset composed of only one observation, displaying high L% and low N%, i.e., the FP subset); (iii) culture-negative/mononuclear cell-predominant (which displayed high L% and low N%); and (iv) culture-positive/phagocyte predominant (which displayed low L% and high N%). Similar biological patterns were observed when only current information was analyzed (**B**).

When prior information was considered, immunologic profiles supported the FN/FP classification: FNs showed the lowest L% (and many FNs displayed lymphocyte values below the lower limit of culture-negative data points), while the FP observation revealed one of the highest L% values among all data points (Fig 7A). FN and FP were also detected when cows previously infected with *E*. *coli* were assessed according to their status at the time of testing, that is, as culture-negative animals (Fig 7B).

**Fig 8:**
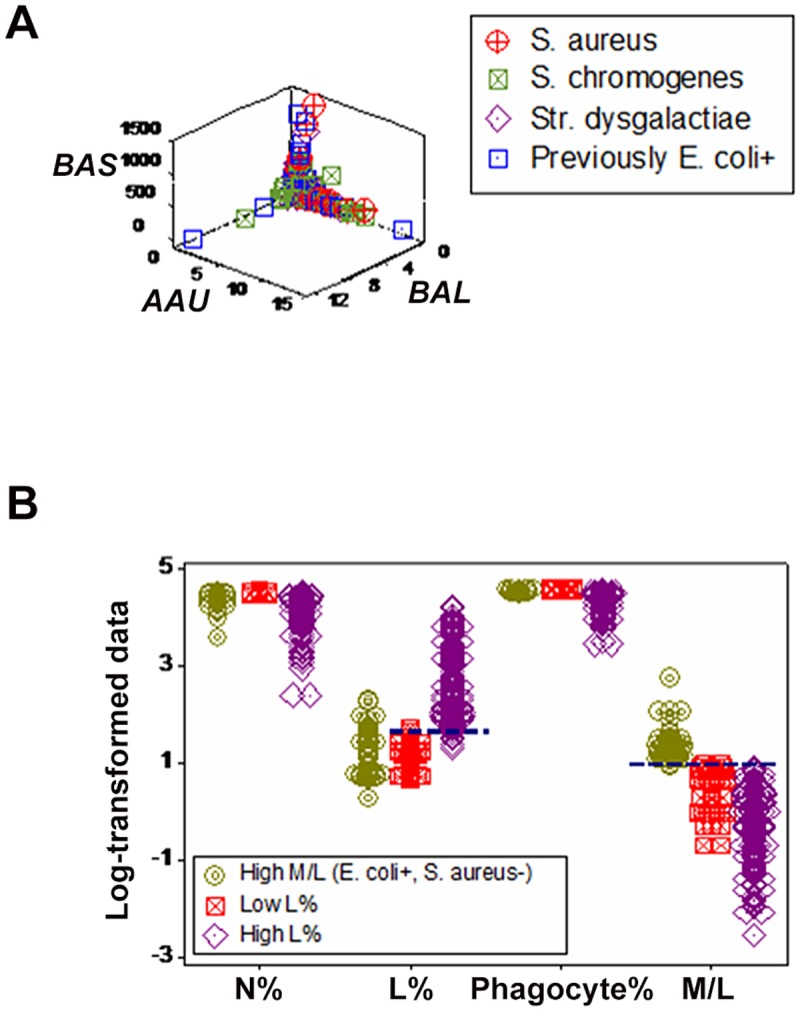
Detection and evaluation of microbial subsets (emergence III). When only culture-positive data were analyzed (including observations collected from cows previously *E*. *coli*-positive), dimensionless indicators distinguished, with perpendicular data inflections, three non-overlapping subsets (**A**). One of such subsets was devoid of *S*. *aureus*+ observations, but predominantly composed of observations collected from previously *E*. *coli*-positive animals. When validated, such subset revealed higher M/L ratio values than the remaining subsets (**B**). The two other subsets differed in their L % values (**B**).

Emergence was also documented when only culture-positive data points were analyzed: three microbial subsets were then detected (Fig 8A). Such subsets were distinguished by monocyte/lymphocyte (M/L) ratio values and/or L percent values (Fig 8B).

**Fig 9:**
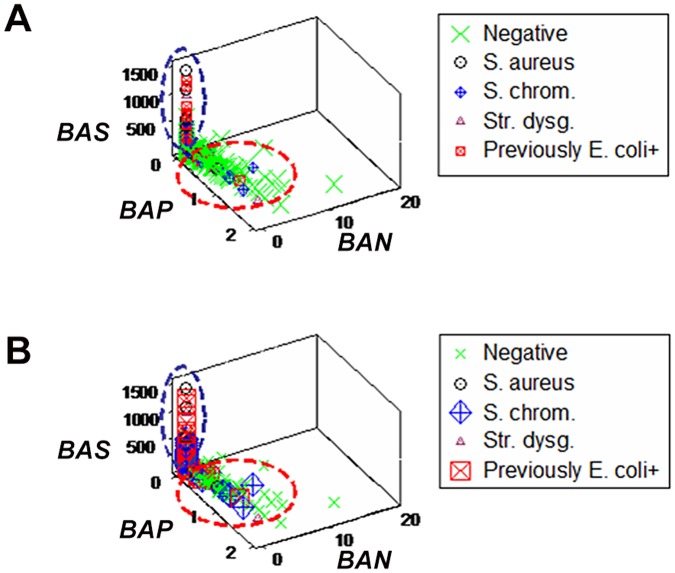
Detection of immuno-microbial subsets (emergence IV). When both culture-negative and -positive patterns were explored with dimensionless indicators, a perpendicular data inflection distinguished two subsets predominantly composed of either culture-positive or—negative observations (vertical and horizontal subsets, respectively **A**). When only the data symbols corresponding to *S*. *chromogenes* and (previously) *E*. *coli*-positive observations were emphasized, most *S*. *chromogenes*-positive observations were found within the horizontal subset—the same subset otherwise revealing culture-negative data points (**B**), while most observations corresponding to animals previously *E*. *coli*-positive were located within the vertical subset (**B**).

When dimensionless indicators were used, both *E*. *coli*- and *S*. *chromogenes*-positive observations revealed a data inflection. Such inflection differentiated two data subsets (Fig 9A and 9B).

**Fig 10:**
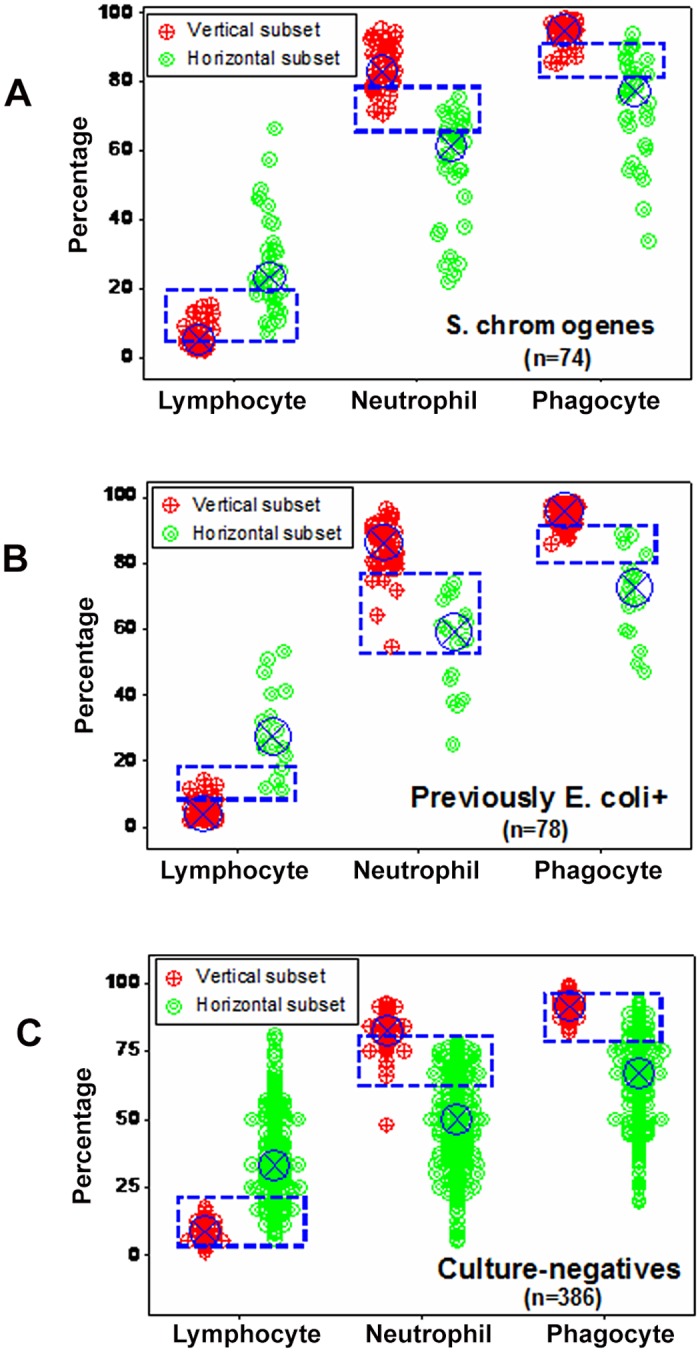
Evaluation of immuno-microbial subsets. When the subsets reported in Fig 9 were explored with leukocyte data, the horizontal subset revealed significantly higher L%, regardless of whether *S*. *chromogenes*-positive only, (previously) *E*. *coli*-positive only, or culture-negative only data were assessed (**A-C**). Similarly, the vertical subset displayed significantly higher N % and phagocyte (P or N+M) percentages than the horizontal subset (*P*<0.001, Mann-Whitney test). Yet, statistical significance did not result in discrimination: overlapping data distributions were found between subsets, in all assessments (**A-C**).

While most *S*. *chromogenes*-positive observations (40/74 or 54%) were found within the horizontal subset (mainly characterized by high lymphocyte percents and negative cultures), most *E*. *coli*-positive data points (61/78 or 78%) were within the vertical subset (characterized by positive cultures and high neutrophil percents, Fig 10A and 10B). Because the same bacteriological tests were used twice, the high N percents associated with negative bacterial cultures (observed 61 times in the later assessment) were not likely to be false (*E*. *coli*-negative) tests. The proportion of *S*. *chromogenes*-positive observations within the horizontal subset was two-fold higher than that of *E*. *coli*-positive observations (45.9 vs. 21.8%, respectively, *P*<0.002, Chis square test). Findings showed two contrasting pictures: leukocyte profiles were similar within subsets (regardless of whether a microbe was isolated or not) but dissimilar across subsets—even when the same microbe was involved. For instance, the median L % for the vertical subset was 4.3 and 5.1 (for *E*. *coli* and *S*. *chromogenes*, respectively) and, for the horizontal subset, it was 27.5 and 22.9% (for *E*. *coli* and *S*. *chromogenes*, respectively). Neutrophil and phagocyte (M and N) median percents revealed similar patterns (Fig 10A and 10B). Yet, the L, N, and phagocyte medians differed across subsets (*P*<0.001, Mann-Whitney test). Culture-negative data points displayed patterns similar to those of *S*. *chromogenes-* and *E*. *coli-*positive groups (Fig 10C). While statistical significance was achieved when subsets were compared, it did not discriminate: a non-trivial number of observations overlapped across subsets (blue boxes, Fig 10A–10C).

**Fig 11:**
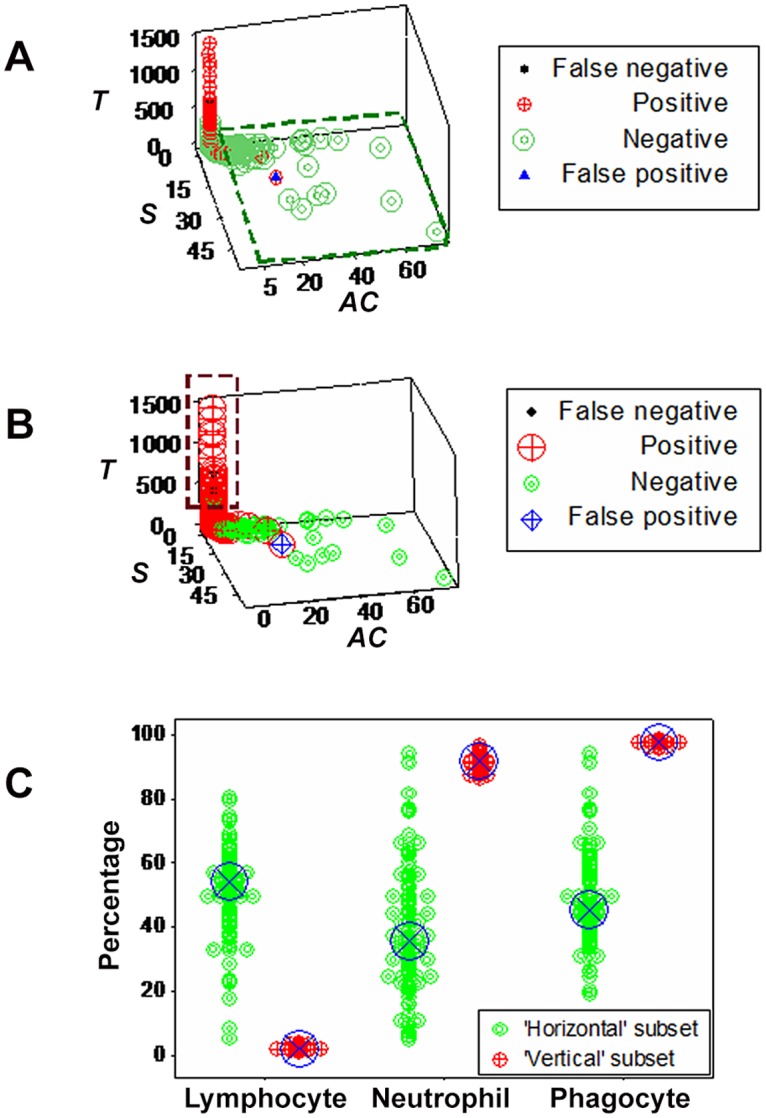
Detection and evaluation of new inflammatory responses (emergence V). Robustness and redundancy were documented: patterns similar to those reported in Fig 9 were obtained when different indicators were utilized, which detected one horizontal subset (mainly composed of culture-negative observations) and one vertical subset (mainly composed of culture-positive observations, **A, B**). The median L, N, and phagocyte percents of such subsets differed, reaching statistical significance (blue circles, *P*<0.001, Mann-Whiney test, **C**).

The sensitivity and specificity of data subsets identified by dimensionless indicators were estimated (Fig 11A and 11B). While the apparent specificity of a culture-negative subset was 91.4% (96/105), because such subset included one FP, its actual value was 92.4% (97/105, Fig 11A). In a subset perpendicular to the previous one, 37 observations were found, of which 34 yielded bacterial cultures (an apparent sensitivity equal to 34/37, or 91.9%). However, because 3 FNs were included, its actual value was 100% (37/37, Fig 11B). Such inferences were biologically validated: higher L% and lower N% were displayed by the specificity subset than by the subset used to estimate sensitivity (Fig 11C). Because this study lacked temporally explicit data, dynamics could not be measured. Because sensitivity and specificity do not account for time and an observational study, such as this, may include several time-related immune responses, the sensitivity and specificity associated with almost ¾ of all data points remained undetermined.

**Fig 12:**
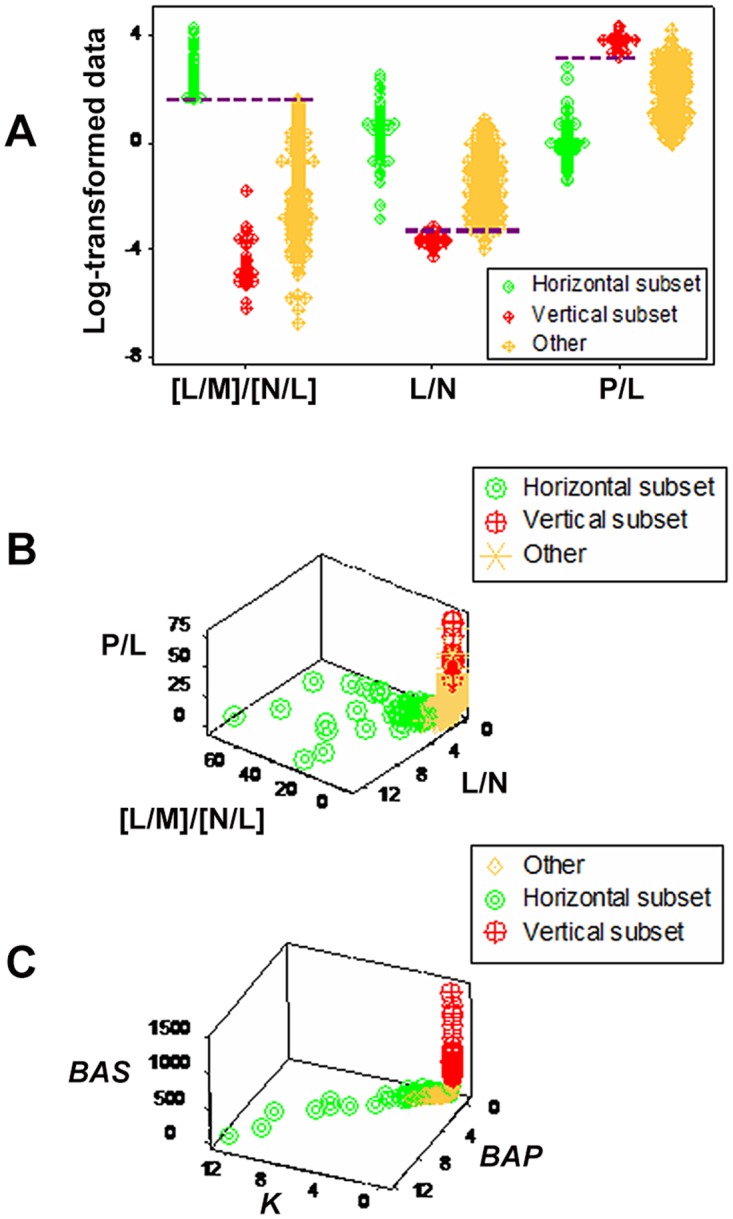
Additional demonstrations of emergence. Non-overlapping leukocyte data distributions differentiated a third (‘other’) subset from the ‘horizontal’ and ‘vertical’ subsets reported in Fig 11 (broken lines, **A**). However, when the leukocyte variables were assessed with a 3D plot, observations of the ‘other’ subset overlapped with the remaining subsets (**B**). That limitation, however, could be overcome if the data were structured as a single line of observations (as shown by an additional set of dimensionless indicators, C) and temporal data were available. When two or more longitudinal observations from the same individual are analyzed within a single (on data point-wide) line, prognosis (and, therefore, diagnosis) can be unambiguously determined, even in the absence of distinct patterns: regardless of any numerical value, arrows connecting pairs of data points can indicate where the most recent data point is coming from/going to, indicating whether such observation approaches the disease-negative or—positive pole of the line.

A double interaction (the [L/M] / [N/L] ratio, Fig 12A) showed a third data subset (here identified as ‘other’) in addition to the ‘horizontal’ and ‘vertical’ subsets observed in Fig 11A and 11B. While partially sharing [L/M] / [N/L] ratio values with the ‘vertical’ subset, the ‘other’ subset differed from the ‘horizontal’ subset and—at least when the L/N ratio was considered—also from the ‘vertical’ subset (Fig 12A). Therefore, at least two PP subsets were distinguished. However, when plotted in 3D space, the leukocyte variables did not show distinct limits: the ‘other’ subset partially overlapped with the remaining ones (Fig 12B). That limitation could have been prevented had the data been structured as a single (one data point-wide) line of observations (as shown in Fig 12C) and temporal data had been available. When two or more longitudinal data points are measured within a single line of observations, an arrow that connects any pair of points will provide *temporal data directionality*, indicating whether the most recent observation is moving toward the disease-negative or -positive pole of the data structure.

## Discussion

This study investigated a novel method that explores host-microbial interactions. To that end, bovine immune responses against intra-mammary infections (IMI) were explored. IMI cause milk production loss and changes in its composition [40–43]. IMI also epitomize diagnostic problems observed across species—humans included. For example, while PCR tests used to detect pathogens in bovine milk seem to possess 100% sensitivity and specificity [44], in sub-clinical cases, the sensitivity of PCR tests decreases substantially [45]. The false-negative rate of milk cultures ranges from 9 to 37% [46]. PCR-related false-positive test results can be due to numerous reasons, such as non-viable bacteria and contamination with extra-mammary bacteria [45]. Errors can also occur when bacterial clearance coexists with protracted inflammation [47].

New or *emergent* patterns were detected when *complex* data structures were generated and *data subsets* were differentiated (Figs 4–12). Because non-structured data did not display such patterns (Figs 1–3), emergence was neither reduced to nor explained by the properties of any one variable; that is, both *irreducibility* and *unpredictability*, in addition to *emergence*, were demonstrated [26–28]. ‘Emergent patterns’ are not truly new: they are so called because such patterns are rarely observed. Such patterns supported two major messages: (i) hidden interactions can be uncovered, provided that the complexity of host-microbial interactions is explicitly explored; and (ii) alone, neither bacteriological tests nor leukocyte profiles are diagnostic.

To detect hidden interactions, a proof-of-concept was developed, which pursued two goals: (i) to increase the number of data shapes to be investigated (so the chances of capturing hidden patterns were increased), and (ii) to prevent an excessively large number of combinations (so the procedure could be conducted in real time). To achieve the first aim, hypothetical (dimensionless) indicators were created and used to uncover data subsets. To validate such subsets, biologically interpretable data were analyzed.

Such approach elicited data points suspected to be false and differentiated immuno- microbial classes, identifying five limitations of bacteriological tests and leukocyte profiles:

- *disease is not ruled out by negative bacterial tests* (e.g., even after antibiotic and anti-inflammatory treatments, culture-negative data of animals previously *E*. *coli*-positive were associated with phagocyte-predominant immune responses);
- *low total cell counts can coexist with phagocyte-rich immune responses;*
- *neither positive bacterial isolation nor high cell counts demonstrate recent disease* (e.g., one *S*. *chromogenes*-positive data subset showed high lymphocyte percents, which displayed values similar to those of culture-negative observations);
- *statistical significance is not synonymous with discrimination* (this study demonstrated, seven times, that comparisons that achieved statistical significance failed to show non-overlapping data distributions); and
- *hidden relationships cannot be detected when simple (non-structured) data formats are used and statistical analyses are performed before data subsets are identified*.

Because two phagocyte-rich data subsets were observed, the classic dichotomy of infection-related classes (the presence of only two [one bacterial-positive and one bacterial-negative] classes) was rejected [48]. Because the observed data subsets were neither cell type- nor bacterium-specific, ‘simple’ models were also rejected [49].

Yet, the analysis of complexity was not complicated: interpretation was achieved without prior knowledge [26, 50]. Because inferences were based on redundancy (Figs 9, 11), interpretations did not depend on any ‘gold standard.’ That is a methodological advantage because, unlike physics (where units of measure can be standardized), in biology, definitions of ‘infection’ and ‘inflammation’ (as well as those on ‘standards’) have not led, so far, to unambiguous and constant interpretations. Because any one biological element (e.g., a cytokine, a cell, a cellular biomarker), over time and in conjunction with other elements may have or perform more than one meaning or function, this pattern recognition-based approach may provide a bio-temporal context to evaluate biomarkers and pharmaceutical drugs. For instance, longitudinal-experimental studies conducted with data structures similar to those here explored could determine the net effect of a drug or the function of a biomarker. By focusing on the complexity and dynamics of immuno-microbial relationships, such studies could elucidate when (in which disease stage) a biomarker is expressed or a drug induces an observable effect. Because complex biological systems (such as diseases) are well-conserved, the temporal (‘early’ or ‘late’) sequence of biological interactions can be assessed regardless of chronological units (hours/days) and not considering whether the tested individual is a slow or fast responder [33]. Because such systems display graphic (three-dimensional) cut-offs, interpretations do not depend on numerical cut-offs—which can induce false-negative and -positive results [18].

Because the labels ‘early’ and ‘late’ immune responses used here were only tentative, longitudinal studies are needed to confirm such temporal classifiers. Such new studies (conducted in non-human and human species) could also explore whether cellular and humoral factors, if simultaneously investigated, increase discrimination.

At least four needs justify future studies on complex host-microbial interactions: (i) *earlier diagnosis*, (ii) *enhanced sensitivity*, (iii) *diminished error rates*, and (iv) *new information*. If computerized, appraisals of such relationships could be more rapidly implemented than bacteriological tests. Complex data structures are more sensitive than classic metrics, e.g., ratios grow larger or faster than percents [34]. When a single line of observations is created, data can change only within a line, facilitating interpretations that do not require confidence intervals. When distinct data patterns are observed, bacterial isolates that induce different immune responses (such as *S*. *chromogenes* isolates) can be distinguished without additional tests.

Because anti-microbial immunity involves, at least, four levels of complexity [51], it is suggested that neither ‘infection’ nor ‘inflammation’ is definable. However, if measured together, both concepts can be described: based on graphic patterns, each data point can be interpreted according to the subset it belongs to. To uncover complex host-bacterial interactions, any data combination can, probably, be used, provided that: (i) the data structure reveals desirable properties (e.g., a single line of observations, distinct patterns, temporal data directionality); (ii) subsets are distinguished and validated; and (iii) statistical tests are conducted after (not before) data subsets are detected. Such propositions may apply to human infections.

## Conclusions

The visualization of leukocyte-bacterial interactions can improve our understanding on infectious diseases. A proof-of-concept that assessed the complexity of such relationships uncovered patterns usually unobserved, which revealed possible solutions to several problems. To explore the dynamics of infectious diseases, additional studies, of longitudinal nature, are needed.

## Supporting Information

S1 Dataset Data analyzed in this study. (XLS)

## References

[1] Gurdasani D, Iles L, Dillon DG, Young E, Elizabeth H, Olson AD, et al (2014) A systematic review of definitions of extreme phenotypes of HIV control and progression. AIDS 28:149–162. 10.1097/QAD.0000000000000049 24149086 PMC3882304

[2] Wynn JL, Wong HR, Shanley TP, Bizzarro MJ, Saiman L, Polin RA (2014) Time for a neonatal-specific consensus definition for sepsis. Pediatr Crit Care Med 15: 523–528. 10.1097/PCC.0000000000000157 24751791 PMC4087075

[3] Segre JA (2013) What does it take to satisfy Koch’s postulates two centuries later? Microbial genomics and *Propionibacteria acnes* . J Investig Dermatol 133:2141–2142. 10.1038/jid.2013.260 23842116 PMC3775492

[4] French GL (2009) Methods for screening methicillin-resistant *Staphylococcus aureus* carriage. Clin Microbiol Infect 15:10–16. 10.1111/j.1469-0691.2009.03092.x 19951329

[5] Wedzicha JA, Brill SE, Allinson JP, Donaldson GC (2013) Mechanisms and impact of the frequent exacerbator phenotype in chronic obstructive pulmonary disease. BMC Med 11:181 10.1186/1741-7015-11-181 23945277 PMC3750926

[6] Casadevall A, Pirofski LA (2003) The damage-response framework of microbial pathogenesis. Nat Rev Microbiol 1:17–24. 10.1038/nrmicro732 15040176 PMC7097162

[7] Deirmengian C, Lonner JH, Booth RE Jr (2005) The Mark Coventry Award—White blood cell gene expression—A new approach toward the study and diagnosis of infection. Clin Orthop Relat Res 440:38–44. 10.1097/01.blo.0000185756.17401.32 16239781

[8] Schuetz P, Christ-Crain M, Muller B (2007) Biomarkers to improve diagnostic and prognostic accuracy in systemic infections. Curr Opin Crit Care 13:578–585. 17762239 10.1097/MCC.0b013e3282c9ac2a

[9] Cook NR (2007) Use and misuse of the receiver operating characteristic curve in risk prediction. Circulation 116: E134–E134. 10.1161/CIRCULATIONAHA.107.715359 17309939

[10] Noble D (2012) A theory of biological relativity: no privileged level of causation. Interface Focus 2 10.1098/rsfs.2011.0067 PMC3262309 23386960

[11] Crofts AR (2007) Life, information, entropy, and time. Complexity 13:14–50. 18978960 10.1002/cplx.20180 PMC2577055

[12] Noble D (2010) Biophysics and systems biology. Phil Trans R Soc A 368: 1125–1139. 10.1098/rsta.2009.0245 20123750 PMC3263808

[13] Villarino AV, Hunter CA (2004) Biology of recently discovered cytokines: discerning the pro- and anti-inflammatory properties of interleukin-27. Arthritis Res Ther 6: 225–233. 10.1186/ar1227 15380038 PMC546292

[14] Dustin ML (2009) The cellular context of T cell signaling. Immunity 30:482–492. 10.1016/j.immuni.2009.03.010 19371714 PMC2905632

[15] Guazzone VA, Jacobo P, Theas MS, Lustig L (2009) Cytokines and chemokines in testicular inflammation: a brief review. Microsc Res Tech 72:620–628. 10.1002/jemt.20704 19263422

[16] Hespel C, Moser M (2012) Role of inflammatory dendritic cells in innate and adaptive immunity. Eur J Immunol 42:2535–2543. 10.1002/eji.201242480 23042650

[17] Becker JM, Rai A, Ringle CM, Völckner F (2013) Discovering unobserved heterogeneity in structural equation models to avert validity threats. MIS Quarterly 37: 665–694.

[18] Cohen J (1983) The cost of dichotomization. Appl Psychol Meas 7:249–253. 10.1177/014662168300700301

[19] Villoslada P, Steinman L, Baranzini SE (2009) Biology and its application to the understanding of neurological diseases. Ann Neurol 65:124–139. 10.1002/ana.21634 19260029

[20] Kurakin A (2009) Scale-free flow of life: on the biology, economics, and physics of the cell. Theor Biol Med Model 6:6 10.1186/1742-4682-6-6 19416527 PMC2683819

[21] Bose B (2013) Systems biology: A biologist’s viewpoint. Prog Biophys Mol Biol 113 358e368 10.1016/j.pbiomolbio.2013.07.001 23872085

[22] Ludewig B, Stein JV, Sharpe J, Cervantes-Barragán L, Thiel V, Bocharov G (2012) A global “imaging” view on systems approaches in immunology. Eur J Immunol 42: 3116–3125. 10.1002/eji.201242508 23255008

[23] Ganusov VV, De Boer RJ (2007) Do most lymphocytes in humans really reside in the gut? Trends Immunol 28:514–518. 10.1016/j.it.2007.08.009 17964854

[24] Shmueli G, Burkom H (2010) Statistical challenges facing early outbreak detection in biosurveillance. Technometrics 52:39–51. 10.1198/TECH.2010.06134 20540788

[25] Cedersund G, Roll J (2009) Systems biology: model based evaluation and comparison of potential explanations for given biological data. FEBS J 276:903–922. 10.1111/j.1742-4658.2008.06845.x 19215297

[26] San Miguel M, Johnson JH, Kertesz J, Kaski K, Díaz-Guilera A, MacKay RS, et al (2012) Challenges in complex systems science. Eur. Phys. J. Special Topics 214:245–271.

[27] Stephan A (2006) The dual role of ‘emergence’ in the philosophy of mind and in cognitive science. Synthese 151:485–498. 10.1007/s11229-006-9019-y

[28] Huneman P (2012) Determinism, predictability and open-ended evolution: lessons from computational emergence. Synthese 185:195–214. 10.1007/s11229-010-9721-7 22486171

[29] Casadevall A, Fang FC, Pirofski LA (2011) Microbial virulence as an emergent property: consequences and opportunities. PLoS Pathog 7(7): e1002136 10.1371/journal.ppat.1002136 21814511 PMC3141035

[30] Yip D, Cho CH (2013) A multicellular 3D heterospheroid model of liver tumor and stromal cells in collagen gel for anti-cancer drug testing. Biochem Biophys Res Commun 433 327–332. 10.1016/j.bbrc.2013.03.008 23501105

[31] Klinke DJ (2009) Validating a dimensionless number for glucose homeostasis in humans. Ann Biomed Eng 37:1886–1896. 10.1007/s10439-009-9733-y 19513847 PMC4402237

[32] Nadell CD, Bucci V, Drescher K, Levin SA, Bonnie LC, Bassler BLC, et al (2013) Cutting through the complexity of cell collectives. Proc. R. Soc. B 280:20122770 10.1098/rspb.2012.2770 23363630 PMC3574390

[33] Rivas AL, Jankowski MD, Piccinini R, Leitner G, Schwarz D, Anderson KL, et al (2013) Feedback-based, system-Level properties of vertebrate-microbial interactions. PLoS ONE 8(2): e53984 10.1371/journal.pone.0053984 23437039 PMC3577842

[34] Fair JM, Rivas AL (2013) Systems Biology and ratio-based, real-time disease surveillance. Transb Emerg Dis (Epub: Sept 11). 10.1111/tbed.12162 24024609

[35] Oliver SP, Gonzalez RN, Hogan JS, Jayarao BM, Owens WE (2004) Microbiological Procedures for the Diagnosis of Bovine Udder Infection and Determination of Milk Quality. 4th ed National Mastitis Council, Verona, WI, USA.

[36] Blum S, Freed M, Zukin N, Shwimmer A, Weissblit L, Khatib N, et al (2010) Bovine subclinical mastitis caused by *Mannheimia granulomatis* . J Vet Diagn Invest 22:995–997. 10.1177/104063871002200627 21088192

[37] Leitner G, Eligulashvily R, Krifucks O, Perl S, Saran A (2003) Immune cell differentiation in mammary gland tissues and milk of cows chronically infected with *Staphylococcus aureus* . J Vet Med B 50:45–52. 10.1046/j.1439-0450.2003.00602.x 12710501

[38] Rivas AL, Quimby FW, Blue J, Coksaygan O (2001) Longitudinal evaluation of bovine mammary gland health status by somatic cell counts, flow cytometry and cytology. J Vet Diagn Invest 13:399–407. 10.1177/104063870101300506 11580061

[39] Bannenberg GL, Chiang N, Ariel A, Arita M, Tjonahen E, Gotlinger KH, et al (2005) Molecular circuits of resolution: formation and actions of resolvins and protectins. J Immunol 174:4345–4355. 15778399 10.4049/jimmunol.174.7.4345

[40] Leitner G, Merin U, Silanikove N (2004) Changes in milk composition as affected by subclinical mastitis in goats. J Dairy Sci 87:1719–1726. 10.3168/jds.S0022-03702(04)73325-1 15453484

[41] Leitner G, Krifucks O, Merin U, Lavi Y, Silanikove N (2006) Interactions between bacteria type, proteolysis of casein and physico-chemical properties of bovine milk. Int Dairy J 16:648–654. 10.1016/j.idairyj.2005.10.020

[42] Leitner G, Merin U, Lavi Y, Egber A, Silanikove N (2007) Aetiology of intramammary infection and its effect on milk composition in goat flocks. J Dairy Res 74:186–193. 10.1017/S0022029906002299 17227594

[43] Merin U, Fleminger G, Komanovsky J, Silanikove N, Bernstein S, Leitner G (2008) Subclinical udder infection with *Streptococcus dysgalactiae* impair milk coagulation, properties: the emerging role of proteose-peptones. Dairy Sci Technol 88:407–419. 10.1051/dst:2008022

[44] Koskinen MT, Holopainen J, Pyörälä S, Bredbacka P, Pitkala A, Barkema HW, et al (2009) Analytical specificity and sensitivity of a real-time polymerase chain reaction assay for identification of bovine mastitis pathogens. J Dairy Sci 92:952–959. 10.3168/jds.2008-1549 19233788

[45] Koskinen MT, Wellenberg GJ, Sampimon OC, Holopainen J, Rothkamp A, Salmikivi L, et al (2010) Field comparison of real-time polymerase chain reaction and bacterial culture for identification of bovine mastitis bacteria. J Dairy Sci 93:5707–5715. 10.3168/jds.2010-3167 21094742

[46] Pitkala A, Gindonis V, Wallin H, Honkanen-Buzalski T (2005) Interlaboratory proficiency testing as a tool for improving performance in laboratories diagnosing bovine mastitis. J Dairy Sci 88:553–559. 10.3168/jds.S0022-0302(05)72717-x 15653520

[47] Blum ES, Heller ED, Leitner G (2014) Long term effects of *Escherichia coli* mastitis. Vet J 201: 72–77. 10.1016/j.tvjl.2014.04.008 24906501

[48] Grimes DA, Schulz KF (2002) Uses and abuses of screening tests. Lancet 359:881–884. 10.1016/S0140-6736(02)07948-5 11897304

[49] Evans MR, Grimm V, Johst K, Knuuttila T, de Langhe R, Lessells CM, et al (2013) Do simple models lead to generality in ecology? Trends Ecol Evol 28:578–583. 10.1016/j.tree.2013.05.022 23827437

[50] Pinsky MR (2010) Complexity modeling: Identify instability early. Crit Care Med 38:S649–S655. 10.1097/CCM.0b013e3181f24484 21164410

[51] Cambi A, Figdor CG (2005) Levels of complexity in pathogen recognition by C-type lectins. Curr Opin Immunol 17:345–351. 10.1016/j.coi.2005.05.011 15950451 PMC7127008

